# Genomic features and fitness cost of co-existence of *bla*_KPC-2_ and *bla*_VIM-2_ plasmids in ICU-derived pan-drug resistant *Pseudomonas aeruginosa*

**DOI:** 10.3389/fcimb.2025.1617614

**Published:** 2025-08-27

**Authors:** Lin Zheng, Zixian Wang, Xin Zhang, Gejin Lu, Jie Jing, Shiwen Sun, Yang Sun, Xue Ji, Bowen Jiang, Lingwei Zhu, Xuejun Guo

**Affiliations:** ^1^ Key Laboratory of Jilin Province for Zoonosis Prevention and Control, Changchun Veterinary Research Institute, Chinese Academy of Agriculture Sciences, Changchun, Jilin, China; ^2^ China-Japan Union Hospital, Jilin University, Changchun, Jilin, China

**Keywords:** carbapenem-resistant *Pseudomonas aeruginosa*, blaKPC-2, blaVIM-2, plasmid, fitness cost

## Abstract

**Backgroud:**

The emergence of carbapenem-resistant *Pseudomonas aeruginosa* (CRPA) co-producing KPC-2 and VIM-2 has increased the healthcare threats.

**Results:**

In this study, a CRPA strain 18102011, was isolated from the bile of a burn patient in ICU of China. Its whole genome was sequenced via the PacBio platform. The molecular characteristics of the genome were analyzed to assess the genetic environment of the carbapenemase genes *bla*_KPC-2_ and *bla*_VIM-2_. Antimicrobial susceptibility, plasmid stability, bacterial growth curves, and plasmid conjugation were measured. Strain 18102011 exhibited a resistant pattern to all 23 antibiotics tested, which could be defined as a pan-drug resistant *P. aeruginosa* strain. Two plasmids were identified in this strain: the Inc_pRBL16_ mega-plasmid pP2011–1 carrying *bla*_VIM-2_ and the IncP6 plasmid pP2011–2 carrying *bla*_KPC-2_. *bla*_VIM-2_ was located in the region of In2057 (a novel class 1 integron) that was inserted into pP2011-1, and the expression of the *bla*_VIM-2_ gene was increased by the PcW_TGN-10_ promoter located at the 5’-CS. For the *bla*_KPC-2_ gene, the core module Tn*3*-IS*Kpn27*-*bla*_KPC_-ΔIS*Kpn6* served as the *bla*_KPC-2_ platform in pP2011-2, and the expression of the *bla*_KPC-2_ gene was achieved via the P1 promoter located downstream of IS*Kpn27*. This expression pattern resulted in MICs of 4,096 μg/mL of imipenem for both strain 18102011 and its transconjugant D2011. Both plasmids were stable in strain 18102011 and could be co-transferred to other strains.

**Conclusion:**

This study raised concerns regarding the high stability and non-inferior fitness of *bla*_KPC-2_-*bla*_VIM-2_-CRPA, shed light on its genomic characteristics, and underscored the importance of continued surveillance of CRPA.

## Introduction

*Pseudomonas aeruginosa* accounts for 10-15% of nosocomial infections worldwide (Blanc et al., 1998). It can attach to the surfaces of medical instruments through biofilm formation, facilitating its spread within hospitals, particularly in the ICU (Cornaglia et al., 2000; Li et al., 2022). Most ICU patients use broad-spectrum antimicrobials to treat bacterial infections, and the prolonged use of antibiotics to eradicate bacterial infections is commonly practiced (Pang et al., 2019). However, the acquisition of antibiotic resistance genes by mobile genetic elements (e.g., plasmids, transposons, integrative elements, and conjugative elements), combined with their transfer among bacterial strains, leads to the development of multidrug-resistant *P. aeruginosa* strains in patients (De Vos et al., 1997; Partridge et al., 2018; Botelho et al., 2019; Allameh et al., 2020; Mohammadnejad et al., 2023). Such infections are associated with high mortality rates, ranging from 18 to 61% (Kang et al., 2003). Although carbapenems are the most important antibiotics for treating their *P. aeruginosa* infections, the bacteria can be resistant to them due in part to the acquisition of carbapenemase genes, complicating treatment (Partridge et al., 2018; Botelho et al., 2019). Some strains even exhibit pan-drug resistance, rendering subsequent treatments with drugs such as colistin and amikacin ineffective, which creates a therapeutic dilemma. Consequently, antimicrobial resistance has become a global health challenge that threatens many medical achievements of the last century and causing serious harm to health system outcomes.

In this study, *P. aeruginosa* strain 18102011 was isolated from the bile of a burn patient in a hospital ICU in 2018 (Changchun, China). The strain was genetically investigated to evaluate the genetic mechanism of its drug resistance. The whole-genome sequence of the strain was generated, and its molecular characteristics were subsequently investigated. The strain belonged to multi-locus sequence typing (ST2374) and serotype O4. Furthermore, the strain co-harbored the *bla*_KPC-2_ and *bla*_VIM-2_ genes, enabling it to resist carbapenems. The MICs of imipenem and meropenem (carbapenem) were both >256 μg/mL. Additionally, it was a pan-drug resistant *P. aeruginosa* that was resistant to 23 antibiotics, including amikacin, colistin, and fosfomycin. Further genetic analyses were applied to the plasmids pP2011–1 carrying *bla*_VIM-2,_ and pP2011–2 carrying *bla*_KPC-2_ to characterize their genetic environments. The transcriptional expression of resistance genes *bla*_KPC-2_ and *bla*_VIM-2_ changed after imipenem exposure. Moreover, the fitness cost, loss of resistance genes under serially passage, and horizontal transmission ability of the *bla*_KPC-2_ and *bla*_VIM-2_ genes in bacteria were analyzed. These results provide a deeper understanding of the acquisition of drug resistance genes in *P. aeruginosa*.

## Materials and methods

### Identification of *bla*_KPC-2_-*bla*_VIM-2_-CRPA

A CRPA obtained from bile collected from a patient in the intensive care unit (ICU) of a public Chinese hospital in 2018, exhibited resistance to both imipenem and meropenem. This isolate was forwarded to our laboratory for species identification and antimicrobial susceptibility testing via the BD Phoenix-100 system, using *Escherichia coli* ATCC25922 as the quality control for susceptibility testing (The antibiotics tested were shown in **Supplementary Table S1**). Additionally, the isolate was stored at -80°C for potential future use. The experimental protocols were approved by the Ethics Committee of the Jilin University (JDKQ202316EC).

To ensure the accuracy of our findings, the MICs of imipenem and meropenem were determined by E-test. Furthermore, the MICs for other antibiotics that have not been tested in the BD Phoenix-100 system, such as colistin, ceftazidime-avibactam, and fosfomycin, which were commonly used to treat carbapenem-resistant bacteria, were also ascertained using E-test (The antibiotics tested were shown in **Supplementary Table S2**). And the quality control strain was *E. coli* ATCC25922. Drug-resistance levels, including resistance, intermediary, and sensitivity thresholds, were assessed by the guidelines established by the Clinical and Laboratory Standards Institute.

It was subject to polymerase chain reaction (PCR) and Sanger sequencing and targets the *oprL* gene (which was specific to *P. aeruginosa*), which encoded a peptidoglycan-associated lipoprotein (De Vos et al., 1997). Additionally, PCR assays were performed to detect the presence of carbapenemase genes, including *bla*_IMP_, *bla*_SPM_, *bla*_AIM_, *bla*_VIM_, *bla*_GIM_, *bla*_BIC_, *bla*_SIM_, *bla*_NDM_, *bla*_DIM_, *bla*_KPC_ and *bla*_OXA-48_ (Poirel et al., 2011) (The primer sequences and PCR conditions were listed in **Supplementary Table S3**).

### Sequencing and genome sequence assembly

Bacterial genomic DNA was isolated using the UltraClean microbial DNA extraction kit (Qiagen, Germany) and sequenced via a PacBio RS II sequencer (Pacific Biosciences, USA). The sequence reads were *de novo* assembled using the SMARTdenovo assembler (http://github.com/ruanjue/smartdenovo). The assembly results were corrected based on sequencing data through three rounds of error correction by using the Racon software (version 1.4.13). Subsequently, three rounds of error correction were performed using the Pilon software (version 1.22) with second-generation reads, yielding the final assembly results.

### Genome annotation and comparison

Precise bacterial species identification was evaluated using pair-wise ANI (http://www.ezbiocloud.net/tools/ani) analysis between the genomes generated in this study and the *P. aeruginosa* reference genome sequence PAO1 (GenBank ID: NC_002516.2). A ≥95% ANI cut-off was used to define bacterial species (Yoon et al., 2017). The PAst (https://cge.food.dtu.dk/services/PAst/) server was used to perform O-antigen classification. In addition, *RAST 2.0* (Brettin et al., 2015) and *BLASTP*/*BLASTN* (Boratyn et al., 2013) searches were used to predict open reading frames (ORFs), whereas comparisons with the ResFinder 4.0 (Bortolaia et al., 2020) (https://cge.cbs.dtu.dk/services/ResFinder/), CARD (https://card.mcmaster.ca) (Alcock et al., 2023) and VFDB (Liu et al., 2022) (http://www.mgc.ac.cn/VFs/) databases were used to identify acquired resistance genes and virulence genes. In addition, the ISfinder (Varani et al., 2011) (https://www-is.biotoul.fr/; Lastest Database Update 2021-9-21), TnCentral (https://tncentral.ncc.unesp.br), INTEGRAL (Moura et al., 2009) (http://integrall.bio.ua.pt/), and ICEberg 2.0 (Liu et al., 2019) (http://db-mml.sjtu.edu.cn/ICEberg/) platforms were used to identify mobile elements. The online database BPROM (Cassiano and Silva-Rocha, 2020) was used for promoter prediction. Pairwise sequence comparisons were conducted using *BLASTN* searches. Functional analysis of proteins in families and domain prediction was conducted using the InterPro (https://www.ebi.ac.uk/interpro) database. Gene organization diagrams were drawn in Inkscape 1.0 (http://inkscape.org/en/).

Multilocus sequence typing (ST) was conducted by evaluating gene sequence data (including seven conserved housekeeping genes: *acsA*, *aroE*, *gtaA*, *mutL*, *nuoD*, *ppsA*, and *trpE*) with the pubMLST platform (https://pubmlst.org/).

### Conjugation experiments

Plasmid transferability was tested using *E. coli* EC600 (rifampicin resistant) and strain 18102011 as the recipient strain and donor strain, respectively. Both the donor and recipient strains were cultured separately overnight at 37°C. Adjust the bacterial concentrations of both recipient strain EC600 and the donor strain 18102011 to 10^9^ CFU/mL. The donor and recipient strains were then subsequently mixed at a 1:1 ratio. The mixture was then spotted onto a 1cm^2^ hydrophilic nylon membrane filter (Millipore; 0.45 µm pore size), which was placed on an LB agar plate and incubated at 37°C for 6 h to initiate mating. After incubation, cells were recovered from the filter and serially diluted from 10–^1^ to 10^-9^, with three parallel replicates per dilution. The dilution was spotted onto LB agar (containing 80 µg/mL rifampicin and 4 µg/mL imipenem) and LB agar (containing 80 µg/mL rifampicin) to select the carbapenem-resistant *E. coli* transconjugants and recipient strain *E. coli* EC600, respectively. The entire experimental procedure was repeated three times. The conjugation transfer efficiency between strain 18102011 and EC600 was calculated using the **Equation (1.1)**. Antimicrobial susceptibility of transconjugants was determined using the BD Phoenix-100 system, while the MIC of imipenem for both strain 18102011 and its transconjugant D2011 was determined by the broth dilution method.


(1.1) $$Conjugation\;transfer\;efficiency\;=\;\frac{Number\;of\;exconjugants\;\left(CFU/mL\right)}{Number\;of\;recipient\;bacteria\;\left(CFU/mL\right)}$$

### Bacterial growth curve assay

In studies assessing plasmid fitness burden, it’s standard and scientifically appropriate to compare an isogenic pair. To specifically determine the fitness cost associated with the presence of plasmids harboring *bla*_KPC-2_ and *bla*_VIM-2_ genes in strain 18102011, bacterial growth curves were constructed for both transconjugant D2011 (carrying *bla*_KPC-2_ and *bla*_VIM-2_) of strain 18102011 and recipient strain *E. coli* EC600. The overnight culture of strain D2011 and EC600 were diluted to 0.5 McFarland standard and subsequently diluted 1:100 in antibiotic-free LB broth. Over the next 12 hours, the optical density (OD_600_) of each culture was monitored at 2-hour intervals via a NanoPhotometer N60 (Implen, Germany). The data represent the mean ± SEM of two independent experiments. Statistical differences were determined by two-tailed *t*-test (*p< 0.05).

### Plasmid stability testing

Strain 18102011 was grown at 37°C in a shaking incubator set to 200 rpm and serially passaged for 10 days, with each passage diluted 1:500 in antibiotic-free LB broth. After 5 and 10 days, the cultures were serially diluted and plated on antibiotic-free LB agar. Fifty single colonies were randomly selected for detection of the *bla*_KPC-2_ and *bla*_VIM-2_ genes by PCR on the 5^th^ and 10^th^ day, respectively, and were used as markers to reflect the loss of their plasmid. The data represent the mean ± SEM of two independent experiments. Statistical differences were determined by two-tailed *t*-test (*p< 0.05).

### RNA preparation and transcriptome sequencing

Bacterial genomic RNA (before and after the addition of imipenem) was isolated using the RNAprep Pure Cell/Bacteria Kit RNAprep Pure (TianGen, China). Starting with total RNA, the mRNA was purified by rRNA depletion. And cDNA libraries were constructed using mRNA. Transcriptome sequencing was performed on triplicate samples, which was carried out by Illumina NovaSeq. Using HTSeq v0.6.1, the number of reads mapped to each gene was counted. FPKM values for each gene were then calculated based on gene length and the corresponding read counts. FPKM, which stands for Fragments Per Kilobase of transcript sequence per Million base pairs sequenced, accounts for both sequencing depth and gene length, making it a widely used method for estimating gene expression levels. The DESeq R package (1.18.0) was employed to determine differential expression of strain 18102011 carrying plasmids before and after the addition of imipenem, using a model based on the negative binomial distribution. Using the Equstion (1.2), we calculate the fold change in expression levels of the resistance genes (*bla*_KPC-2_ and *bla*_VIM-2_), conjugative transfer genes, and replicon genes, before and after antibiotic treatment. The resulting *p*-values were adjusted using the Benjamini-Hochberg method to control the false discovery rate. Genes with an adjusted *p*-value<0.05, identified by DESeq, were classified as differentially expressed.


(1.2) $$log2FoldChange\;=log2\frac{antibiotic\;treated\;group}{antibiotic-free\;group}$$

### Quantitative real-time polymerase chain reaction

All primers and probes for *bla*_KPC-2_ gene, *bla*_VIM-2_ gene, and *repA* genes (Inc_pRBL16_ and IncP6) were designed using Primer 5.0 software, based on the sequences from strain 18102011 carrying plasmid 1 (CP116229) and plasmid 2 (CP116230) (The primer sequences are listed in **Supplementary Table S4**). DNA contamination was removed from RNA samples using the 4x gDNA wiper rMix (reaction conditions: 42°C for 2 min). cDNA was then synthesized using the used 5x HiScripII qRT SuperMIx II. The reverse transcription program included incubation at 50 °C for 15 min, followed by incubation at 85 °C for 5 s. Quantitative real-time polymerase (qPCR) analysis specifically targeted four key genetic elements. These included the *bla*_KPC-2_ gene and *bla*_VIM-2_ gene responsible for encoding KPC-2 and VIM-2 carbapenemases respectively. The analysis also targeted the Inc_pRBL16_ *repA* and IncP6 *repA* genes, which encode essential replication initiation proteins for plasmid 1 and plasmid 2 (The qPCR conditions were listed in **Supplementary Table S5**). The experiment was conducted with three technical replicates using three-fold serial dilutions of cDNA. The entire experimental procedure was repeated three times.

### Nucleotide sequence accession numbers

The complete sequences of strain 18102011, plasmid pP2011-1, and plasmid pP2011–2 have been submitted to GenBank under the accession numbers CP116228, CP116229, and CP116230, respectively. The transcriptome data of strain 18102011 and its plasmids before and after the addition of imipenem have been uploaded to the China National Center for Bioinformatics (CNCB) (http://ngdc.cncb.ac.cn/gsub/) under accession number CRA023298.

## Results and discussion

### Identification and antimicrobial resistance profile of strain 18102011

After strain 18102011 was cultured overnight at 37°C on Brain Heart Infusion (BHI) agar containing an imipenem concentration of 4 μg/mL, distinct colonies with smooth and regular edges, measuring approximately 0.5 mm in diameter, were observed. These colonies exhibited non-fusion growth, and pyocyanin production, and were devoid of metallic sheens. Subsequently, strain 18102011 was identified as *P. aeruginosa* using the BD Phoenix-100 automated identification system and sequencing of the *oprL* gene, a marker specific to this species. Following this identification, a comprehensive analysis of its drug resistance spectrum was conducted, revealing resistance to all evaluated antibiotics (**Supplementary Tables S1**, **S2**).

### Genomic characterization of the *P. aeruginosa* strain 18102011

To characterize the genome of the bacterium thoroughly, we employed Single Molecule Real-Time (SMRT) sequencing. The genome sequencing data revealed a 6.6 Mb chromosome in strain 18102011 (GenBank ID: CP116228), exhibiting a GC content of 66.2%. Additionally, the presence of two plasmids was detected in this bacterium. This strain’s genome revealed an ANI value of ≥95% with the reference strain *P. aeruginosa* PAO1 (GenBank ID: NC_002516.2), verifying the species information for this bacterium again.

The molecular genetic characteristics of the bacterium were analyzed to determine whether it was a member of a potential epidemic clone group. The isolate belonged to the multi-locus sequence type ST2374 and serotype O4 based on MLST and PAst identification, respectively. As of March 2024, the pbMLST database contained two total strains of ST2374 *P. aeruginosa*, including strain SX69 from a sputum origin in China and strain 127Gr isolated from soft tissue in Belarus in 2016. More than twenty serotypes of *P. aeruginosa* were known, of which serotype O4 was not known as a multidrug-resistant serotype (Nasrin et al., 2022). However, serotype switching from O4 to the multidrug-resistant serotype O12 had been observed under specific conditions (Thrane et al., 2015).

Three virulence genes were detected on the chromosome of strain 18102011: the phospholipase C (PLC) gene (*plcH*) and exotoxin genes (*exoS* and *exoT*). PLC was a thermolabile hemolysin that degrades phospholipid surfactants and reduces surface tension, thus preventing alveoli from collapsing completely when air leaves them during breathing (Khan et al., 2021; Wang et al., 2021). The ExoS and ExoT proteins could be secreted via the type III secretion system (T3SS) and could disrupt the cytoskeleton, induce host cell rounding, disrupt intercellular tight junctions, prevent wound healing, and inhibit bacterial internalization into epithelial cells and macrophages (Rao et al., 2021; Jouault et al., 2022). Consistent with previous reports, the isolate harboring *exoS*-like sequences did not contain *exoU*-like sequences (Feltman et al., 2001). In this study, in addition to the *exoU* gene encoding ExoU (a T3SS effector), the *exoA* gene encoding Exotoxin A (ExoA), which was also a strong pathogenic virulence factor such as ExoU, was not identified (Jørgensen et al., 2005; Foulkes et al., 2019; Morgan et al., 2021). Additionally, the *exoY* gene encoding ExoY (another T3SS effector) was also not detected in this study. It might exhibit greater cytotoxicity to epithelial cells than strains secreting active ExoY, as ExoY had been found to possibly play a protective role at certain stages of bacterial infection, either to facilitate host colonization or to establish and/or maintain chronic infection in the host (Silistre et al., 2021). Thus, this strain might cause cellular damage in immunocompromised patients, but it didn’t cause acute toxicity.

According to the Resfinder database, CARD database, and PCR results, the bacteria carried the acquired genes *bla*_VIM-2_ and *bla*_KPC-2_ simultaneously. Furthermore, through the Resfinder platform, other acquired resistance genes were identified in strain 18102011, including aminoglycoside resistance genes (*aph(3’)-IIb*, *aac(6’)-Ib-cr*, *ant(2’’)-Ia*), β-lactam resistance genes (*bla*_OXA-396_, *bla*_PAO_, *bla*_PER-1_), a sulfonamide resistance gene (*sul1*), a chloramphenicol resistance gene (*catB7*), a quinolone resistance gene (*qnrVC6*), and a fosfomycin resistance gene (*fosA*). These resistance genes were inserted into chromosomes and plasmids by mobile elements. Plasmid-mediated drug resistance genes could lead to their rapid spread among bacteria. Pan-resistance *P. aeruginosa* co-carrying *bla*_VIM-2_ and *bla*_KPC-2_ genes was first reported in Colombia and had subsequently spread in this country, harboring many resistance genes (Correa et al., 2015; Rada et al., 2021), which was not common in China.

### Overview of plasmid pP2011–1 carrying *bla*_VIM-2_

The bacteria carried two plasmids, which were named pP2011–1 and pP2011-2, in this study. The mega-plasmid pP2011-1 (GenBank ID: CP116229) carrying *bla*_VIM-2_ was 474 kb and exhibited a GC content of 56.9%. The plasmid harbored a *repA* (replication initiation) gene sharing ≥96% nucleotide identity to *repA*_IncpRBL16_. The Inc_pRBL16_ plasmid was first reported in the mega-plasmid p12969-DIM (GenBank ID: KU130294) of strain *P. aeruginosa* 12969 (Sun et al., 2016). As of March 2024, there were many genomes containing *repA*_IncpRBL16-like_ sequences in the GenBank database, all of which belonged to *Pseudomonas* spp. However, the pP2011–1 plasmid had a low genetic identified with the previously reported Inc_pRBL16_ family plasmid (Jiang et al., 2020; Dong et al., 2022), and there were large structural variations. The Inc_pRBL16_ family plasmids carried by bacteria may have co-evolved with their chromosomes to maintain their presence in the host bacteria.

**Figure 1:**
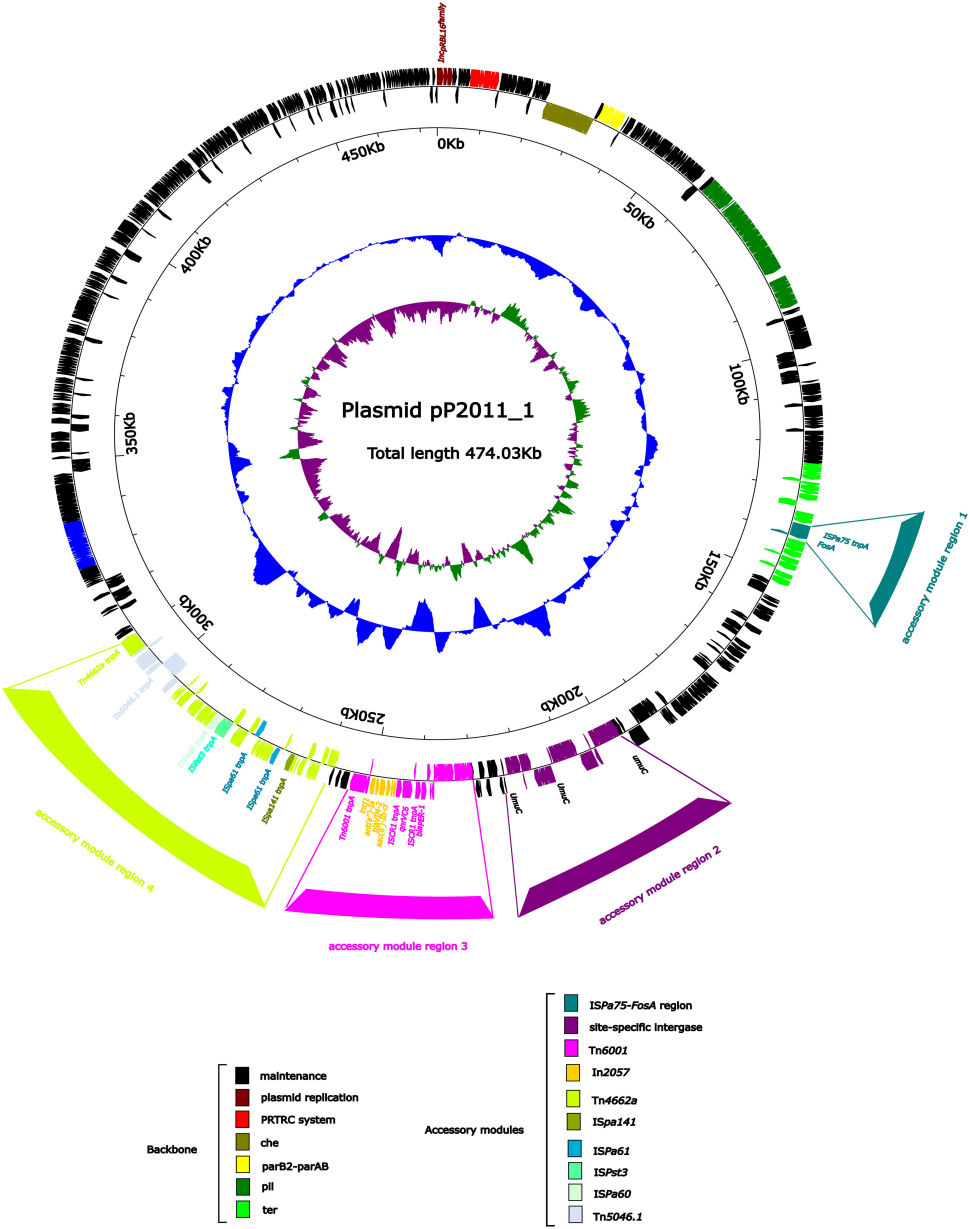
Annotation of the plasmid pP2011-1. The circles represented (from outside to inside) the following: (1) Genome functional annotation. The backbone region was black, the plasmid replicon was brown, the PRTRC system was red, the *che* region was dark green, the parB2-parAB region was yellow, the *pil* region was lighter green, and the *ter* region was bright green. The IS*Pa75* and *fosA* genes form the accessory module 1 region (dark cyan), whereas the *dnaK* and *yaeT* genes, along with several phage integrase genes and stress protein genes, form the accessory module 2 region (purple), which was inserted into the UmuC region (purple). The accessory module 3 region (pink) comprises Tn*6011* (pink) and In2057 (orange). The accessory module 4 region (green-yellow) comprises IS*pa141* (olive), IS*pa61* (dark blue), IS*pst3* (turquoise), IS*Pa60* (honeydew), Tn*4662a* (green-yellow) and Tn*5046.1* (alice blue); (2) GC skew calculated as [(G-C)/(G+C)]; and (3) GC content.

As shown in **Figure 1**, the backbone of plasmid pP2011–1 contained genes for partitioning (*parB2-parAB*), conjugal transfer (*cpl* and *tivF*), chemotaxis (*che*), pilus assembly (*pil*), and tellurium resistance (*ter*), in addition to *repA*_IncpRBL16_ (**Figure 1**). The plasmid also contained 3 novel recombination sites in addition to the common recombination site 2 for Inc_pRBL16_ family plasmids (Jiang et al., 2020). IS*Pa75* (IS*66* family) and *fosA* formed the accessory module 1 region that was inserted between two hypothetical proteins of the tellurium resistance region. In addition, *dnaK* and *yaeT*, along with several phage integrase genes and stress protein genes, form the accessory module 2 region that was inserted into the region of the catalytic subunit of Pol V (UmuC), which was a major recombination site of Inc_pRBL16_ family plasmids. During conjugation, plasmids were transferred as single-stranded DNA, which in turn activates the bacterial SOS stress response. The SOS response coordinated the expression of dozens of bacterial genes involved in DNA repair and cell cycle control, and it was known to fuel bacterial evolvability through an increase in recombination and mutagenesis (Rodríguez-Beltrán et al., 2021). The accessory module 3 region comprised heavy-metal efflux protein genes (*merE*, *merD*, *merA*, *merP*, *merT*, and *merR*), Tn*6001* (Tn*3* family), two copies of IS*CR1* (IS*91* family), *bla*_PER-1_, and *qnrVC6*, and a novel class 1 integron In2057 carrying *ant(2’’)-IIa*, *bla*_VIM-2_ and *aac(6’)-Ib-cr*, which was inserted between the hypothetical protein genes that were 708 bp and 1,257 bp in length. Finally, the accessory module 4 region included IS*Pa141* (IS*30* family), IS*Pa61* (IS*L3* family), IS*Pst3* (IS*21* family), IS*Pa60* (IS*As1* family), Tn*4662a* (Tn*3* family), and Tn*5046.1* (Tn*3* family), which were inserted between the hypothetical protein and phage protein genes.

### Overview of plasmid pP2011–2 carrying *bla*_KPC-2_

**Figure 2:**
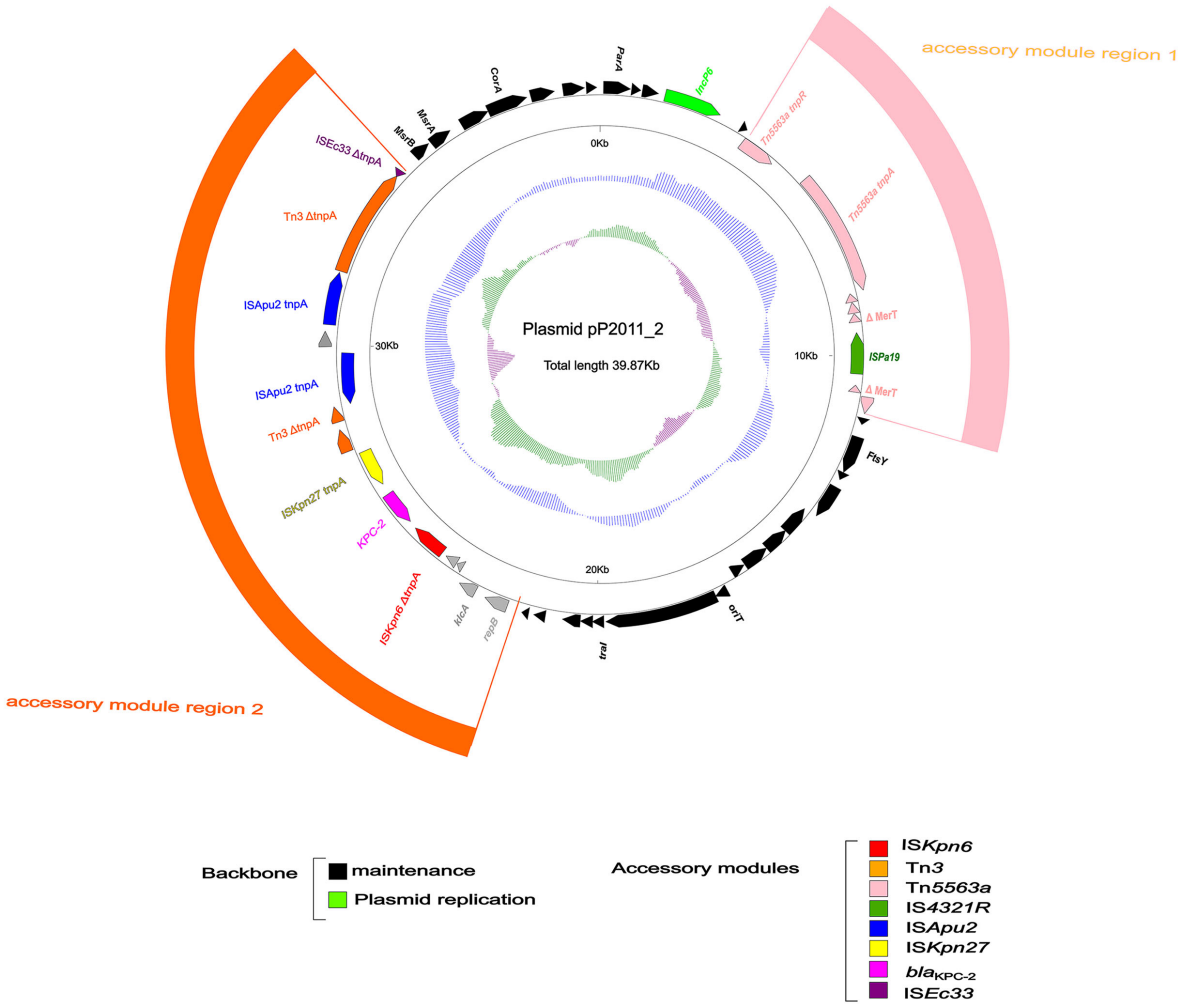
Annotation of plasmid pP2011-2. The circles represented (from outside to inside) the following: (1) Genome functional annotation. The backbone region was black, in which the plasmid replicon was green. Tn*5563a* (pink) and IS*Pa19* (sea-green) comprise the accessory module 1 region (pink), whereas the module 2 region (orange) comprises IS*Kpn6* (red), *bla*_KPC-2_ (magenta), IS*Kpn27* (yellow), Tn*3* (orange), IS*Apu2* (blue), and IS*Ec33* (purple); (2) the GC skew was calculated as [(G-C)/(G+C)]; and (3) the GC content.

As shown in **Figure 2**, the pP2011–2 plasmid (GenBank ID: CP116230) carrying *bla*_KPC-2_ was 40 kb in length and exhibited a GC content of 58.1%. The plasmid harbored a *repA* gene sharing ≥96% nucleotide identity to *repA*_Incp6_. The IncP6 plasmid was first identified in pRms149 (GenBank ID: GCA_019400855.1) of the *P. aeruginosa* JX05 strain in 2005 (Haines et al., 2005). According to the GenBank database, this replicon sequence could be found in Enterobacteriaceae, *P. aeruginosa*, and *Aeromonas* strains. The backbone of the *repA*_Incp6_ family plasmid included *repA*_Incp6_, genes for partitioning (*parABC*), and a mobilization region (*mobABCDE*), in addition to two accessory modules in this study. The accessory module 1 region comprised Tn*5563a* (Tn*3* family) and IS*Pa19*, whereas accessory module 2 includes IS*Ec33*-Tn*3*-IS*Apu2*-IS*Apu2*-*orf7*-IS*Kpn27*-*bla*_KPC-2_-ΔIS*Kpn6-korC-orf6-klcA-*Δ*repB*. Typical transposons Tn*4401* and Tn*1722*, both members of Tn*3* family, have been demonstrated to mobilize the *bla*_KPC-2_ at high transposition frequencies (Song et al., 2024). KPC-1/KPC-2 was first identified in *Klebsiella pneumoniae* in 2001 (Yigit et al., 2001). While KPC carried by Enterobacteriaceae, it has also been detected in non-Enterobacteriaceae due to horizontal gene transfer, such as *P. aeruginosa*. Tn*4401* is a removable element that commonly carries the *bla*_KPC-2_ gene in Europe, Brazil, and the United States. In Asia, *bla*_KPC-2_ is mainly located on different variants of Tn*1722*-like transposons (Song et al., 2024). Zhang DF et al. found that there were three divergent forms of *bla*_KPC-2_ transposon unit in *K. pneumoniae*, including Tn*1721*-*bla*_KPC-2_ transposon, IS*26*-Tn*1721*-*bla*_KPC-2_ transposon and IS*26*-*bla*_KPC-2_ transposon (Zhang et al., 2022). The IncP6 plasmid (p10265-KPC) was first characterized in 2016, different from the Tn*1722/*Tn*1721*-like unit transposons (pKP048 from *K. pneumoniae*), the IS*Apu1*-*orf7*-IS*Apu2* structure truncates the Tn*3* transposase and inserts a truncated *bla*_TEM-1_ gene downstream of IS*Kpn27* (Dai et al., 2016). In this study, the distinction lines in the fact that p10265-KPC has a truncated *bla*_TEM-1_ gene inserted between IS*Kpn27* and the *bla*_KPC-2_ gene (Dai et al., 2016).

### Conjugative transfer and resistance phenotype dissemination of *bla*_VIM-2_ and *bla*_KPC-2_-encoding plasmids

To evaluate the transferability of plasmids harboring *bla*_KPC-2_ and *bla*_VIM-2_, conjugation assays were performed by co-culturing the strain 18102011 with *E. coli* EC600. Conjugants grown on dilution gradients of 10^-1^, 10^-2^, and 10^-3^. And recipient bacteria can grow on all dilution gradients from 10–^1^ to 10–^9^ on a medium containing only rifampicin. The conjugation transfer efficiency was calculated to be 10–^6^ using the **Equation (1.1)**. Because the IncP6 plasmid lacked a conjugation-transfer region and only had a mobile region, the plasmid could only carry out horizontal transfer under the synergistic action of the conjugation plasmid Inc_pRBL16_ (Coluzzi et al., 2022). In this study, all suspected transconjugants were picked from the culture medium at 10^3^ dilution gradients, with number of 19, 15, and 21 respectively (corresponding to three parallel replicates). A single carbapenem-resistant gene, namely *bla*_KPC-2_ or *bla*_VIM-2_, was not found by conjugation assays. Although the MICs of transconjugant D2011 for many antibiotics tested decreased significantly, indicating susceptibility, it was still resistant to imipenem and meropenem (**Supplementary Tables S1**, **S2**). The MICs of strain 18102011 and its transconjugant D2011 against imipenem were determined by broth method, both of which were 4,096 μg/mL. Therefore, the main resistance mechanism of strain 18102011 to carbapenem antibiotics was plasmid-mediated *bla*_VIM-2_ and *bla*_KPC-2_ genes, resulting in a stable carbapenem-resistant transmission phenotype.

### Plasmid stability and fitness cost analysis of *bla*_KPC-2_ and *bla*_VIM-2_-co-harboring strain 18102022

**Figure 3:**
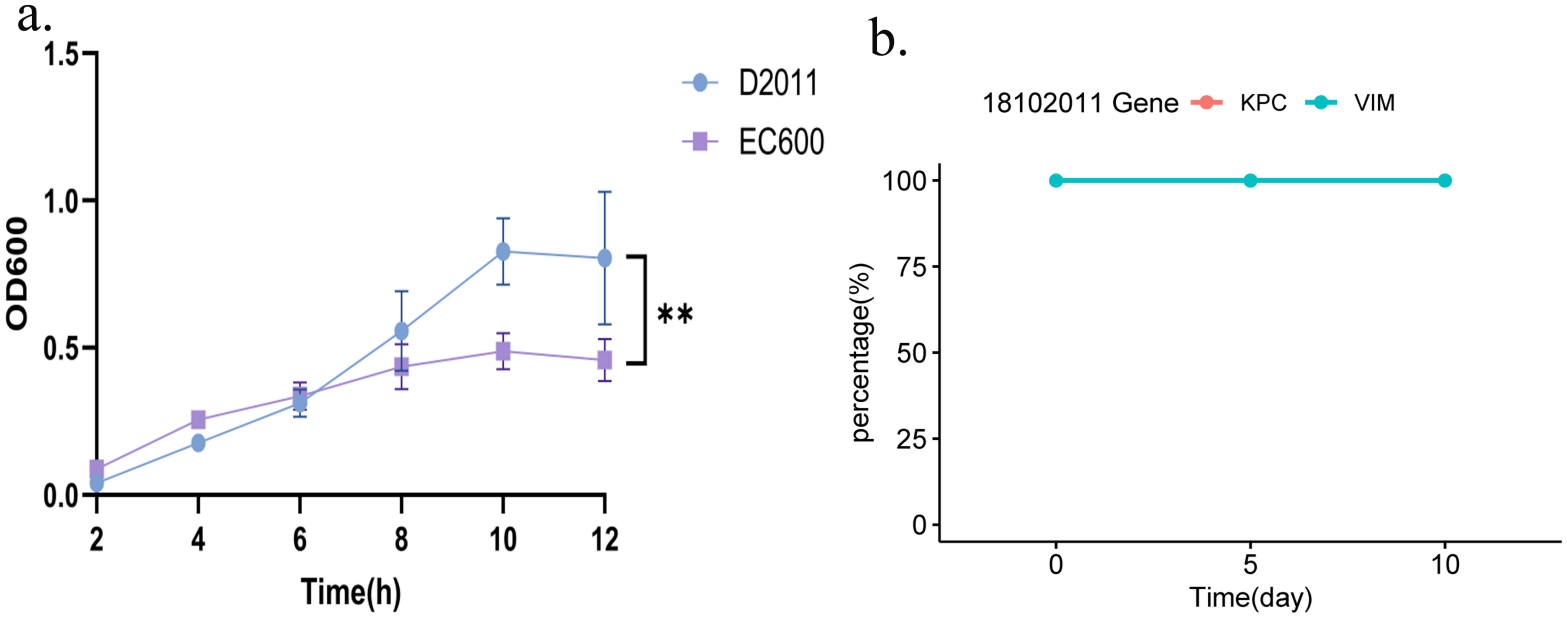
Growth curves of strain D2011 and stability of the *bla*_KPC-2_ or *bla*_VIM-2_ gene. **(a)** Growth curve of strain D2011 carrying the *bla*_KPC-2_ and *bla*_VIM-2_ genes and *E*. *coli* EC600. **(b)** Stability of the *bla*_KPC-2_ and *bla*_VIM-2_ genes during the 10-day serial passage. *p*< 0.05 *(significant), *p*< 0.01 ** (highly significant).

The impact of the acquisition of both plasmids carrying *bla*_KPC-2_ and *bla*_VIM-2_ on the biological fitness cost was evaluated. Notably, the OD_600_ of recipient strain *E. coli* EC600 was significantly lower than that of the transconjugant D2011 (carrying *bla*_KPC-2_ and *bla*_VIM-2_) in 12h. Taken together, these microbiological characteristics indicated that strain 18102011 could carry both the *bla*_KPC-2_ and *bla*_VIM-2_ genes without compromising their fitness and maintaining them stably over time (**Figure 3a**). Furthermore, during serial passage in the laboratory for 10 days to evaluate the stability of plasmids carrying *bla*_KPC-2_ and *bla*_VIM-2_ from strain 1810211. Interestingly, both the *bla*_KPC-2_ and *bla*_VIM-2_ plasmids were present in 50 colonies (100%, 50/50) on the 5^th^ and 10^th^ day, respectively, indicating high stability (**Figure 3b**, PCR identification of *bla*_KPC-2_ and *bla*_VIM-2_ genes of the strain D2011 on 5^th^ and 10^th^ day were shown in **Supplementary Figure S1**). This increased our concern about *bla*_KPC-2_-*bla*_VIM-2_-CRPA, as it posed not only significant challenges in treatment but also a remarkable ability to stably maintain these plasmids without apparent fitness costs, making it difficult to retrogress.

### Transcriptional regulation of *bla*_VIM-2_ and *bla*_KPC-2_ under carbapenem pressure

The integron In2057-*bla*_VIM-2_ sequence was uploaded into the BPROM database, revealing only one promoter with the -10/-35 region (AGCTTACCA/TGTCCA) located within In2057’s integrase. This promoter sequence matched the PcW_TGN-10_ promoter sequence identified in the research by Nesvera J et al., driving the expression of the downstream gene cassette (Nesvera et al., 1998). It had been reported that the TGN-10 motif increased the PcW promoter strength efficiency 15-fold (Jové et al., 2010). Therefore, this promoter might have increased the expression of a series of downstream gene cassettes in this study, including *bla*_VIM-2_. This might be one of the reasons for the high resistance of bacteria to carbapenem.

The IS*Kpn27*-*bla*_KPC-2_ sequence was uploaded into the BPROM database. Only one promoter with the -10/-35 region (ATGTAA/GGATTA) was identified between the IS*Kpn27* and *bla*_KPC-2_ genes. This promoter sequence was consistent with the P1 promoter sequence commonly found in IS*Kpn7*-*bla*_KPC-2_, which drove the expression of the downstream *bla*_KPC-2_ gene (Huang et al., 2019; Huang et al., 2020).

**Figure 4:**
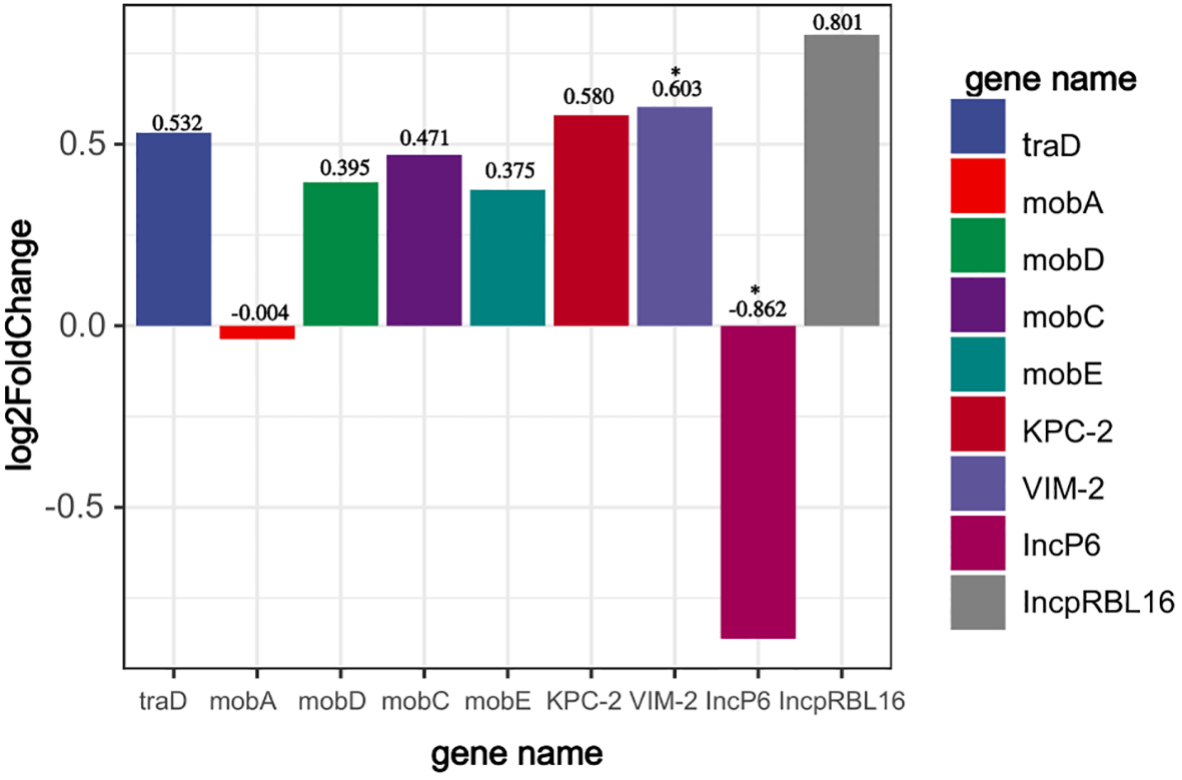
Read counts of the Inc_pRBL16_ (*repA*), IncP6 (*repA*), *bla*_VIM-2_, *bla*_KPC-2_, conjugative transfer genes (*tra*), and mobile genetic genes (*mob*) in strain 18102011. The horizontal axis represents gene name, whereas the vertical axis represents change in read counts (FPKM) of genes after imipenem addition (expressed as log_2_FoldChange). *p*<0.05 *(significant), *p*<0.01 **(highly significant).

**Figure 5:**
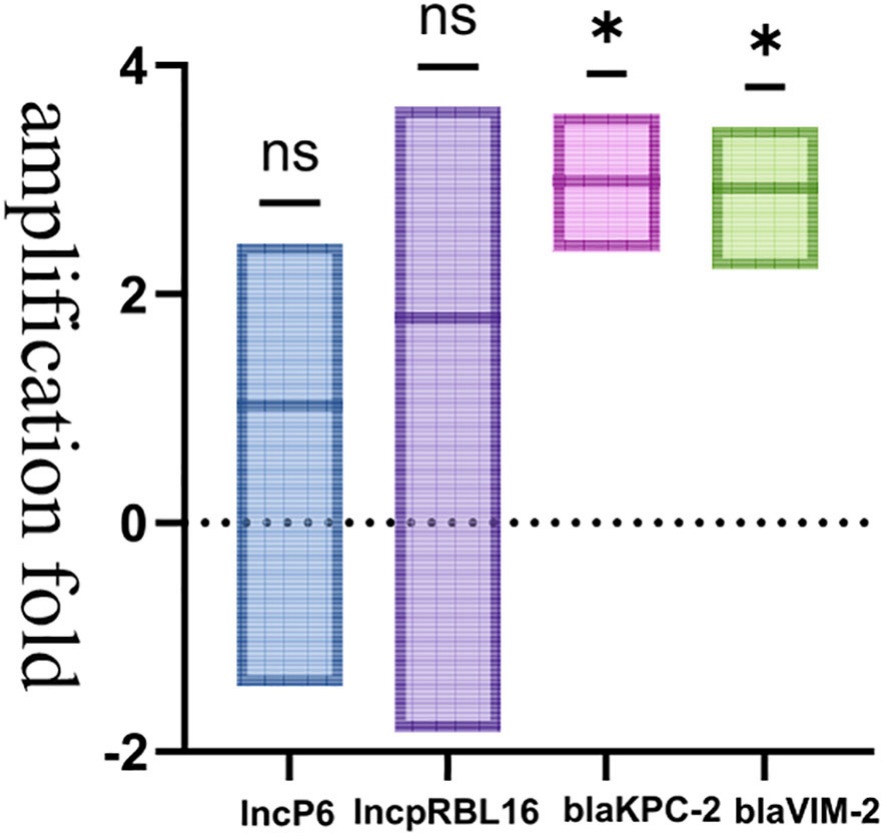
Results of qPCR identification of the Inc_pRBL16_ (*repA*), IncP6 (*repA*), *bla*_VIM-2_, and *bla*_KPC-2_ in strain 18102011. The horizontal axis represents group classifications (IncP6; Inc_pRBL16_; *bla*_KPC-2_; *bla*_VIM-2_), whereas the vertical axis represents amplification fold. *p*<0.05 *(significant), *p*<0.01 **(highly significant), *p*>0.05 ns (not significant).

Through transcriptome analysis of the expression of the *bla*_VIM-2_ gene carried by plasmid pP2011–1 and the *bla*_KPC-2_ gene carried by plasmid pP2011-2, it was found that both genes were up-regulated when imipenem was added to the culture medium (**Figure 4**, the data for **Figure 4** can be found in **Supplementary Table S6**). However, after the addition of imipenem, the expression level of *bla*_VIM-2_ significantly increased, which might be related to the regulatory effect of its upstream strong promoter (**Figures 4**, **5**, the data for **Figure 4** can be found in **Supplementary Table S7**). Additionally, under the influence of imipenem, the expression levels of some other genes on the plasmid also changed. For example, the expression levels of plasmid conjugative transfer gene (*tra*), the mobilization genes (*mob*, excluding *mobA*), and the *repA* gene on the IncpRBL16 plasmid all showed increase, although this increase was not significant (**Figures 4**, **5**). The low levels of read counts (FPKM) for the replicon genes *repA* (located on the Inc_pRBL16_ plasmids) and the conjugative transfer gene (*traD*) indicated their low expression levels (**Supplementary Table S6**). Since the *repA* gene is a key factor for initiating plasmid replication, its low expression leads to a reduction in plasmid copy number. This could be a strategy employed by the Inc_pRBL16_ plasmid to decrease its fitness cost to the host bacterium by maintaining a lower copy number (**Figure 4**).

## Conclusion

Currently, there were relatively few reports on pan-resistant *P. aeruginosa* co-carrying *bla*_VIM-2_ and *bla*_KPC-2_ genes in China, which were mostly concentrated in Columbia. The limited existing reports also lacked an analysis of the fitness costs associated with the plasmids carried by these bacteria. In this study, the presence of *bla*_KPC-2_ and *bla*_VIM-2_ plasmids didn’t cause fitness costs to the growth of the host bacteria. Since the IncP6 plasmid lacked a conjugation transfer region, its horizontal transfer required the assistance of Inc_pRBL16_ conjugation plasmids for co-transfer into *E. coli* EC600. Through horizontal plasmid transfer, high-level resistance to imipenem was stably maintained within the bacterial population. However, there were differences in the composition of the cell membranes between *P. aeruginosa* and *E. coli*, causing the pan-drug resistant phenotype to not be shared between the donor and recipient bacteria through plasmid conjugation transfer. As the treatment of pan-drug resistant bacteria was extremely challenging, their epidemiological and molecular genetics across the world should be closely monitored.

## Data Availability

Publicly available datasets were analyzed in this study. This data can be found here: The complete sequences of strain 18102011, plasmid pP2011-1, and plasmid pP2011-2 have been submitted to GenBank under the accession numbers CP116228, CP116229, and CP116230, respectively. The transcriptome data of strain 18102011 and its plasmids before and after the addition of imipenem have been uploaded to the China National Center for Bioinformatics (CNCB) (http://ngdc.cncb.ac.cn/gsub/) under accession number CRA023298.

## Supplementary material

Supplementary Figure 1 PCR identification of *bla*_KPC-2_ and *bla*_VIM-2_ genes of strain 18102011 on the 5^th^ and 10^th^ day. (a) PCR identification of *bla*_KPC-2_ and *bla*_VIM-2_ genes of strain 18102011 on the 5^th^ day; (b) PCR identification of *bla*_KPC-2_ and *bla*_VIM-2_ genes of strain 18102011 on the 10^th^ day; (c) PCR identification of *bla*_KPC-2_ and *bla*_VIM-2_ genes of strain 18102011 on the 5^th^ day (repeat); (d) PCR identification of *bla*_KPC-2_ and *bla*_VIM-2_ genes of strain 18102011 on the 10^th^ day (repeat).
