# Supplementary material for: Genomic features and fitness cost of co-existence of bla KPC-2 and bla VIM-2 plasmids in ICU-derived pan-drug resistant Pseudomonas aeruginosa
Source: Front Cell Infect Microbiol. 2025 Aug 27;15:1617614. doi: 10.3389/fcimb.2025.1617614 (PMC12420198; doi:10.3389/fcimb.2025.1617614)
Supplement: Supplementary Figure 1 — PCR identification of bla KPC-2 and bla VIM-2 genes of strain 18102011 on the 5th and 10th day. (a) PCR identification of bla KPC-2 and bla VIM-2 genes of strain 18102011 on the 5th day; (b) PCR identification of bla KPC-2 and bla VIM-2 genes of strain 18102011 on the 10th day; (c) PCR identification of bla KPC-2 and bla VIM-2 genes of strain 18102011 on the 5th day (repeat); (d) PCR identification of bla KPC-2 and bla VIM-2 genes of strain 18102011 on the 10th day (repeat). [file DataSheet1.pdf]

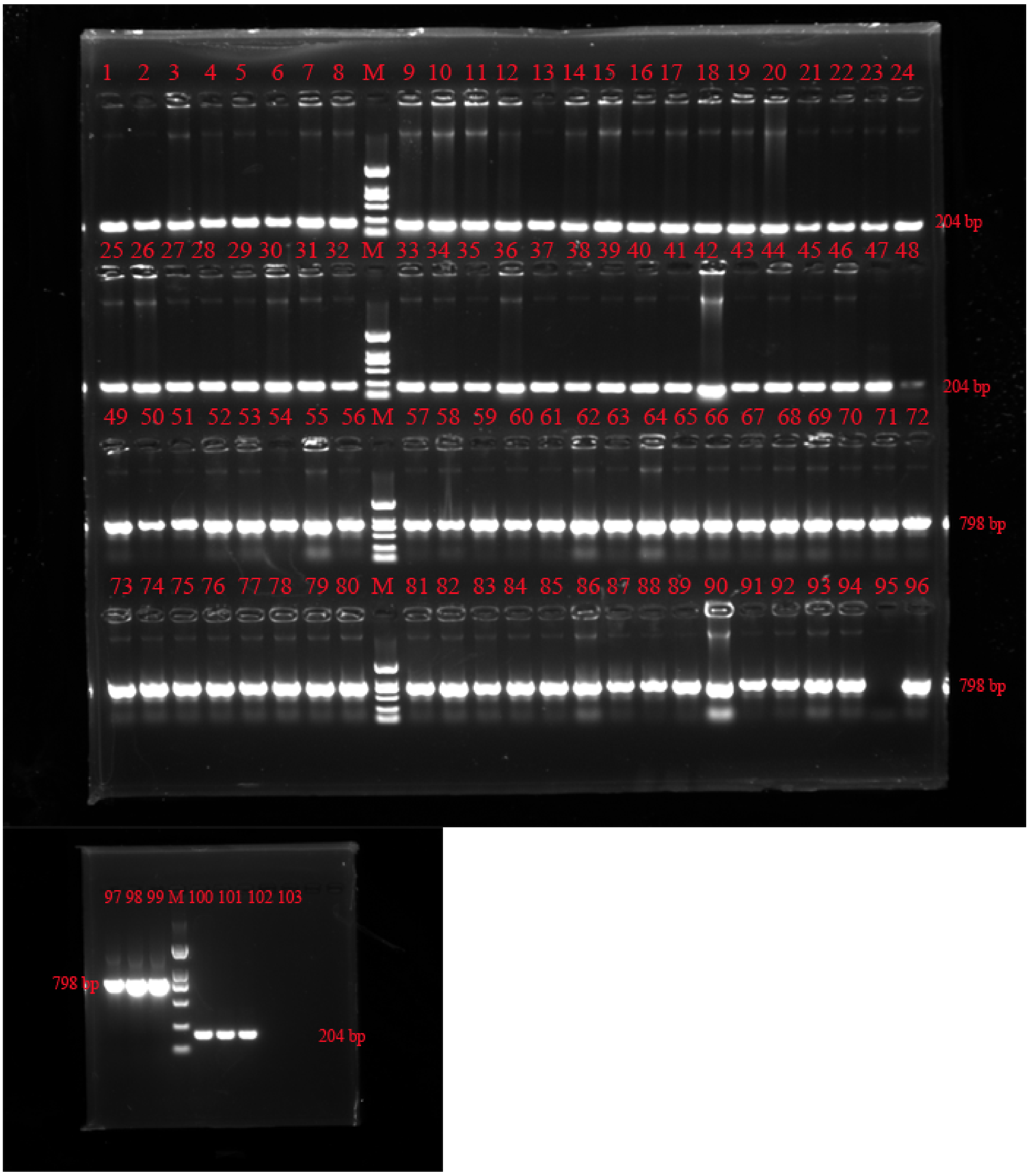

(a) PCR identification of *bla*$_{KPC-2}$ and *bla*$_{VIM-2}$ genes of strain 18102011 on the 5th day. The length of *bla*$_{KPC-2}$ gene was 798 bp, and the length of *bla*$_{VIM-2}$ gene was 204 bp.

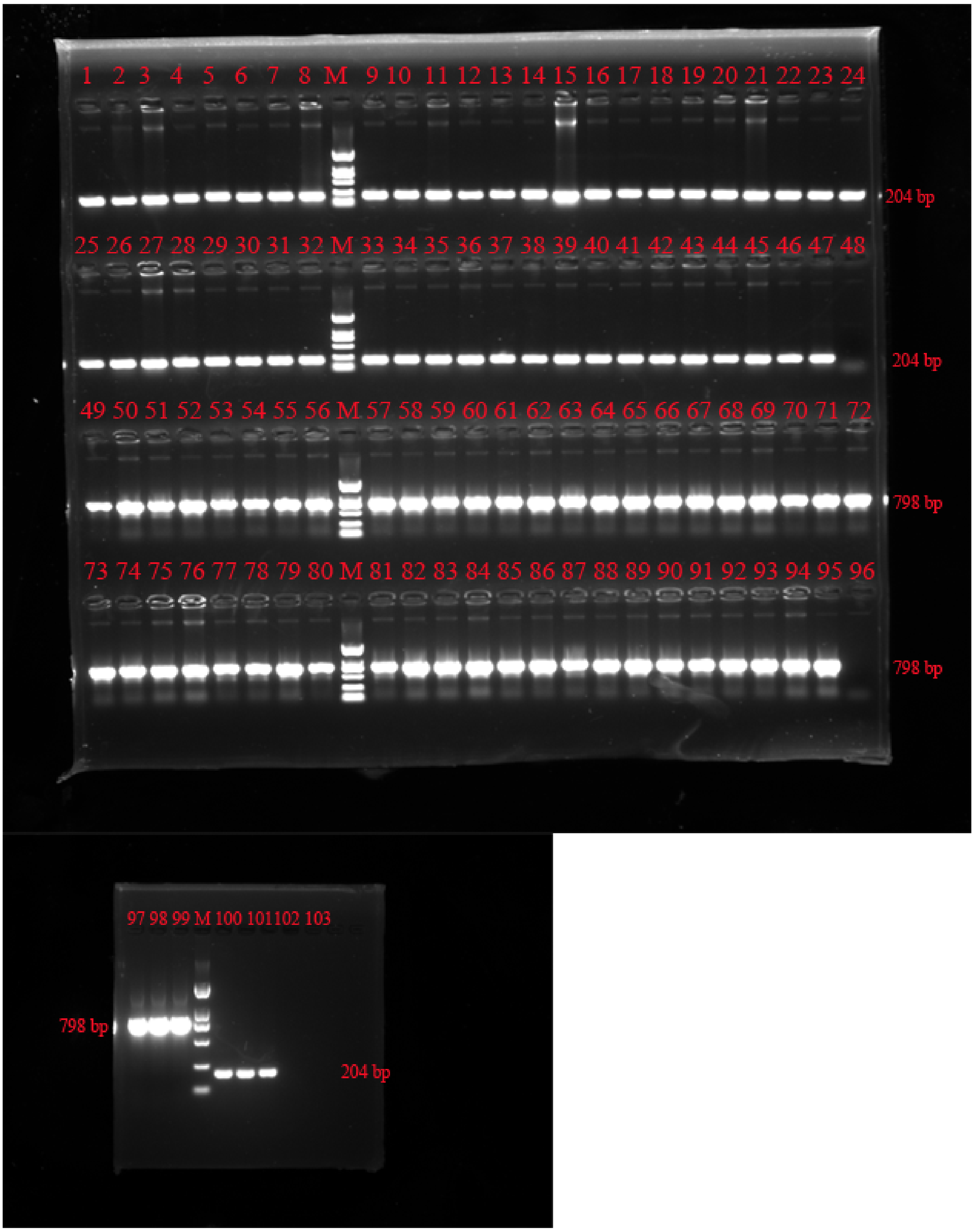

(b) PCR identification of $\textit{bla}_{\text{KPC-2}}$ and $\textit{bla}_{\text{VIM-2}}$ genes of strain 18102011 on the 10th day. The length of $\textit{bla}_{\text{KPC-2}}$ gene was 798 bp, and the length of $\textit{bla}_{\text{VIM-2}}$ gene was 204 bp.

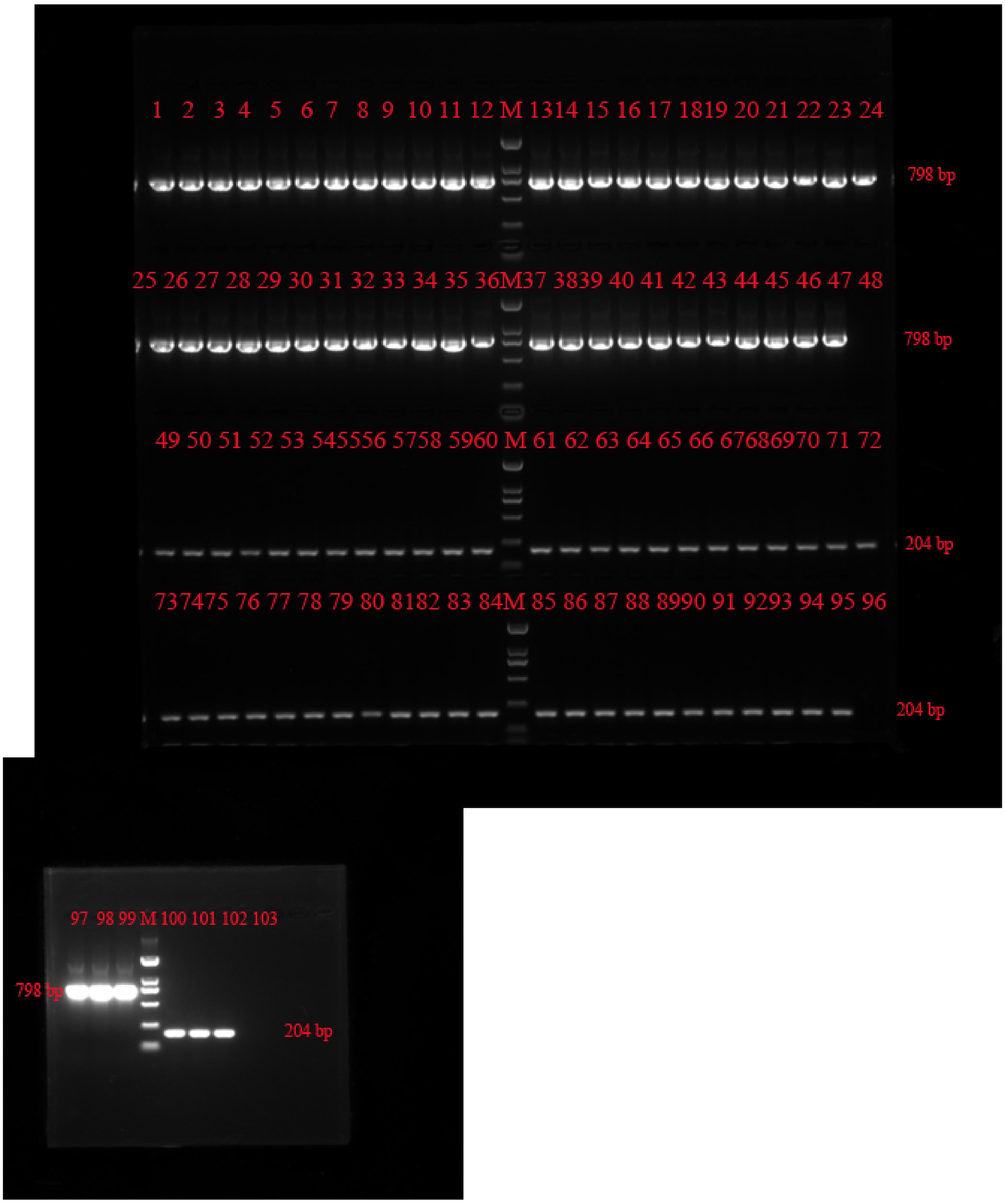

(c) PCR identification of *bla*$_{KPC-2}$ and *bla*$_{VIM-2}$ genes of strain 18102011 on the 5th day (repeat). The length of *bla*$_{KPC-2}$ gene was 798 bp, and the length of *bla*$_{VIM-2}$ gene was 204 bp.

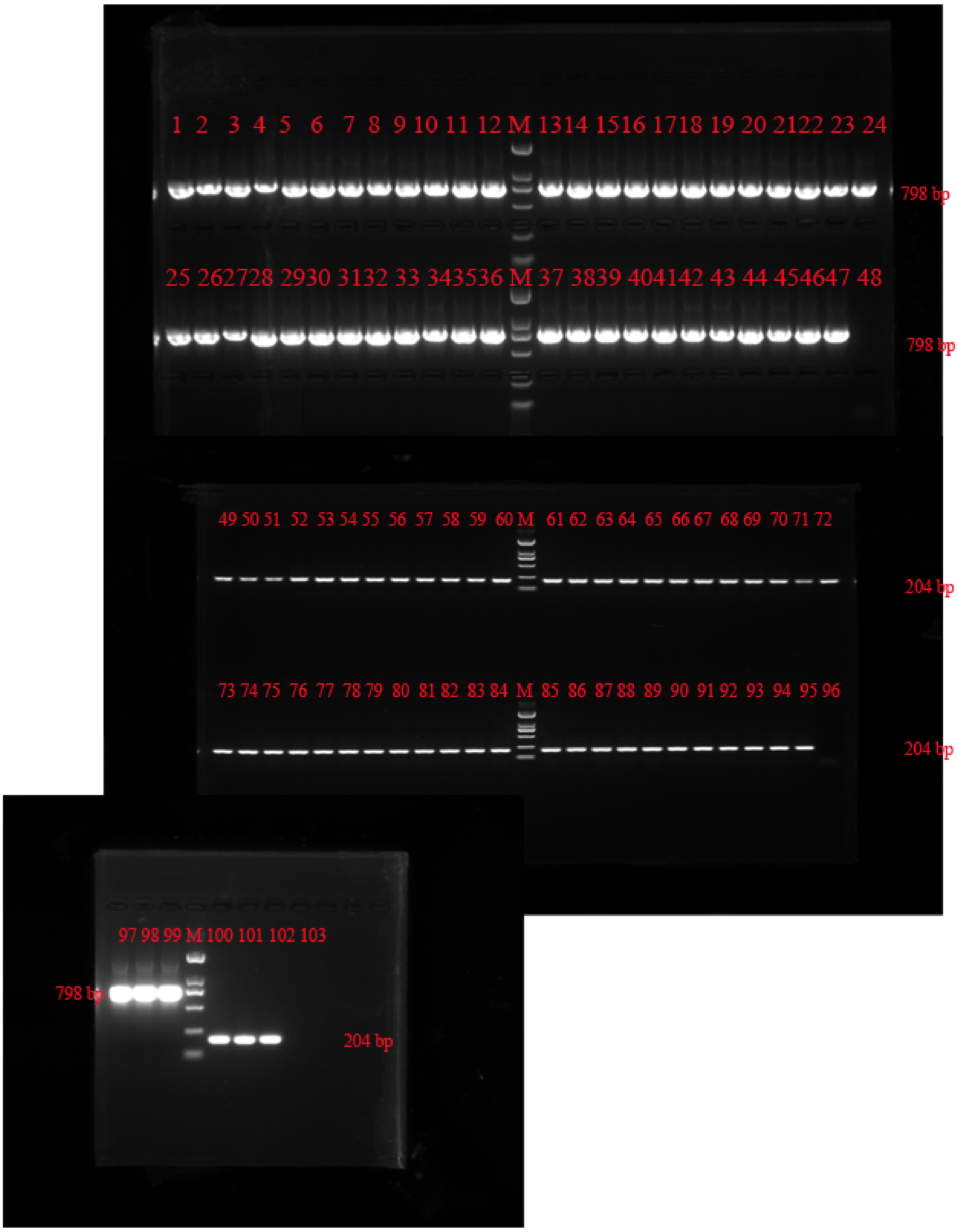

(d) PCR identification of *bla*$_{KPC-2}$ and *bla*$_{VIM-2}$ genes of strain 18102011 on the 10th day (repeat). The length of *bla*$_{KPC-2}$ gene was 798 bp, and the length of *bla*$_{VIM-2}$ gene was 204 bp.
